# Suicidality and self-injury behaviours due to the use of Novel Psychoactive Substances (NPS)

**DOI:** 10.1192/j.eurpsy.2023.161

**Published:** 2023-07-19

**Authors:** L. Orsolini

**Affiliations:** Unit of Clinical Psychiatry, Department of Neurosciences/DIMSC, Polytechnic University of Marche, Ancona, Italy

## Abstract

**Abstract:**

Over the past twenty years a large number of new psychoactive substances (NPS) have entered and modified the recreational drug scene. Their intake has been associated with health-related risks, especially so for vulnerable populations such as people with severe mental illness, who might be at higher risk of suicidality or self-injurious behavior. The consumption and frequent poly-consumption of NPS result in death, suicide, serious self-injury behaviours as well as adverse effects on medical and mental health. Hence, the talk will deal with current data on suicidality and self-injury behaviours due to the use of NPS, particularly considering the suicide and self-injury risk due to the NPS intake among vulnerable people with preexisting severe mental illness.

**Disclosure of Interest:**

None Declared

